# Supplementary material for: Hierarchical Prediction and Perturbation of Chromatin Organization Reveal How Loop Domains Mediate Higher‐Order Architectures
Source: Adv Sci (Weinh). 2025 Jul 8;12(37):e04799. doi: 10.1002/advs.202504799 (PMC12499379; doi:10.1002/advs.202504799)
Supplement: Supplementary file 1 — Supporting Information [file ADVS-12-e04799-s001.pdf]

ADVANCED SCIENCE
Open Access

Supporting Information

for *Adv. Sci.*, DOI 10.1002/advs.202504799

Hierarchical Prediction and Perturbation of Chromatin Organization Reveal How Loop Domains Mediate Higher-Order Architectures

Jiachen Wei, Yue Xue and Yi Qin Gao**

Supplementary information

Properties	Akita	DeepC	Orca	C.Origami	HiCGen
Architecture	CNN	CNN	CNN	Transformer	SwinT
Maximum window size	1 Mb	1 Mb	256 Mb	2 Mb	32 Mb
Highest resolution	2 kb	5 kb	4 kb	8 kb	1 kb
Multiscale outputs	-	-	√	-	√
Generalizable to other cells	-	-	-	√	√
Epigenetic perturbation	-	-	-	√	√

Supplementary Table.1 | Comparison of the capabilities of models to predict genomic interactions.

Cell type	Hi-C	ATAC-seq	CTCF ChIP-seq
GM12878	ENCSR916MFV	ENCSR095QNB	ENCSR000AKB
IMR90	ENCSR345VTI	ENCSR200OML	ENCSR000EFI
HCT116	ENCSR477GZK	ENCSR872WGW	ENCSR240PRQ
HCT116$^{\Delta MED14}$	ENCSR343ELD	ENCSR998BBI	ENCSR446FOM
HCT116$^{\Delta CDK7}$	ENCSR129NAE	ENCSR874GXS	ENCSR341OQG
HCT116$^{\Delta CTCF}$	ENCSR669VZH	ENCSR260SWI	ENCSR160GVO
Sigmoid colon	ENCSR123ZFD	ENCSR846VLJ	ENCSR925GDS
PC3	ENCSR038TZA	ENCSR499ASS	ENCSR359LOD
Prostate gland	ENCSR557QRO	ENCSR999NKW	ENCSR829HTO
MCF7	ENCSR660LPJ	ENCSR422SUG	ENCSR000DMO
Breast epithelium	ENCSR326YHP	ENCSR654UYP	ENCSR304XUZ
PANC-1	ENCSR584RBV	ENCSR591PIX	ENCSR203QEB

Supplementary Table.2 | Accession number of intact Hi-C data, ATAC-seq and CTCF ChIP-seq profiles used for training and evaluating the model.

Cell type	H3K4me1	H3K27ac	H3K4me3	H3K27me3
GM12878	ENCSR000AKF	ENCSR000AKC	ENCSR057BWO	ENCSR000AKD
IMR90	ENCSR831JSP	ENCSR002YRE	ENCSR087PFU	ENCSR431UUY
HCT116	ENCSR161MXP	ENCSR661KMA	ENCSR333OPW	ENCSR810BDB
HCT116$^{\Delta CTCF}$	ENCSR803TBJ	ENCSR671XCL	ENCSR098CPV	ENCSR295BJE
Sigmoid colon	ENCSR812POE	ENCSR268ZCF	ENCSR900UIP	-
PC3	ENCSR566UMF	ENCSR826UTD	ENCSR275NCH	ENCSR881TWJ
Prostate gland	ENCSR642CSX	ENCSR763IDK	ENCSR748RBT	-
MCF7	ENCSR493NBY	ENCSR752UOD	ENCSR985MIB	ENCSR761DLU
Breast epithelium	ENCSR553IAW	ENCSR034ZKE	ENCSR416AUW	ENCSR770WSE

Supplementary Table.3 | Accession number of other epigenetic signals used for analyzing data.

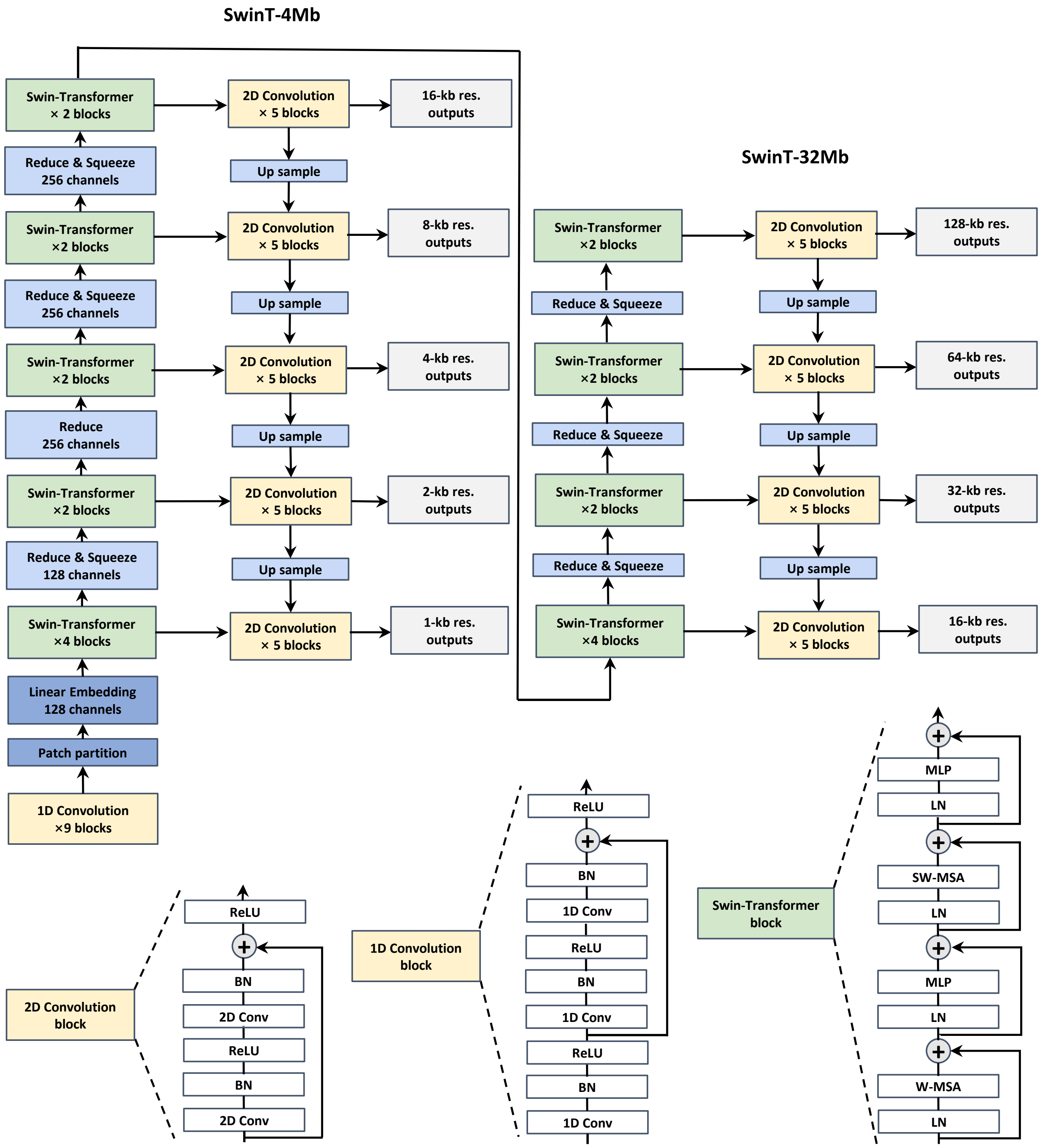

Supplementary Fig.1 | Detailed architecture and blocks of HiCGen. The 1D Convolution module acts as encoder for both sequence and epigenetic signals. The Swin-Transformer module splits the output of encoder into patches with window size=32, and learns each window hierarchically. A series of cascading 2D Convolution modules as decoders are prepared for each resolution. Each decoder takes the diagonalized output of Swin-Transformer module at given resolution and the prediction from the upper-level decoder as input. The model was trained in two stages. SwinT-4Mb was trained in the first stage. SwinT-32Mb was trained in the second stage, with fixed model weights for modules trained in stage-one. BN, LN, MSA and SW-MSA are short for batch normalization, layer normalization, multi-head self-attention and shifted window-based MSA, respectively.

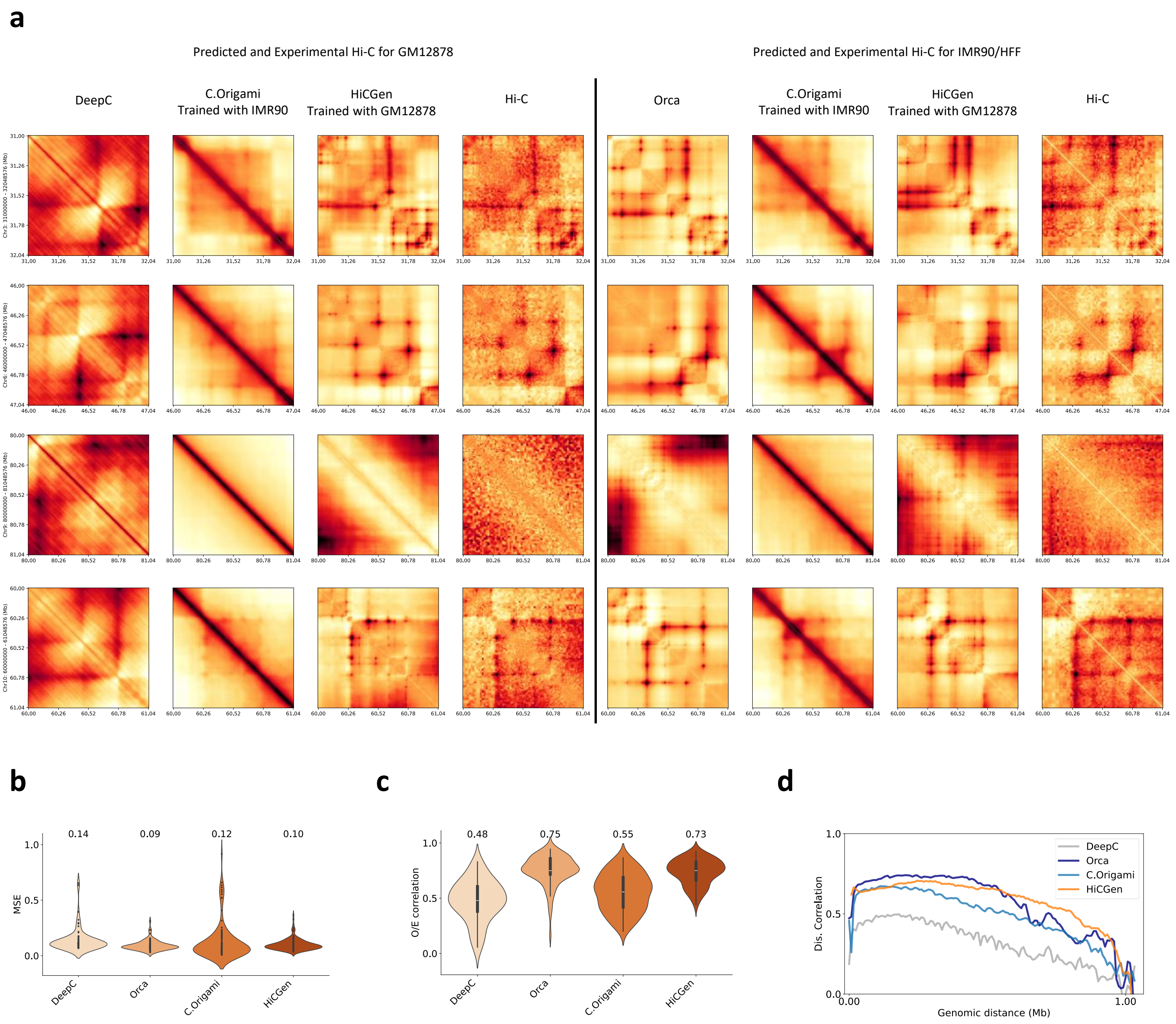

Supplementary Fig.2 | Comparisons of model performance on predicting 1-Mb window of contacts. a, Randomly selected examples of cell-type-specific predictions of 1-Mb contact maps from DeepC, Orca, C.Origami and HiCGen. **b,c,** Comparisons of mean squared error (**b**) and Pearson correlation coefficients between experimental and predicted contacts (**c**) at 8-kb resolution for test sets. **d,** Distance-stratified correlations between experimental and predicted matrices for test-set chromosomes of each model.

a

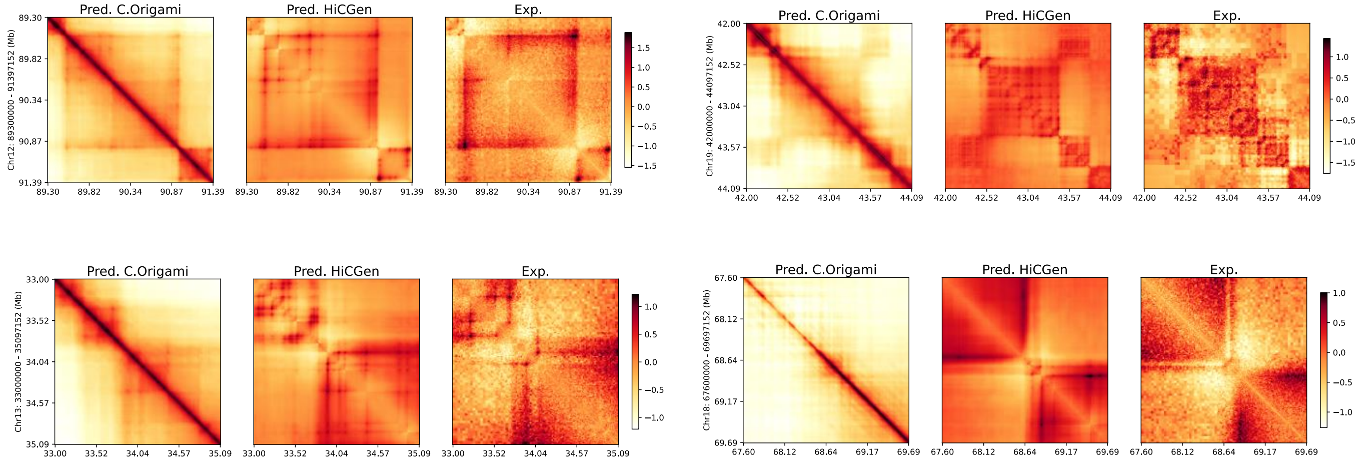

b

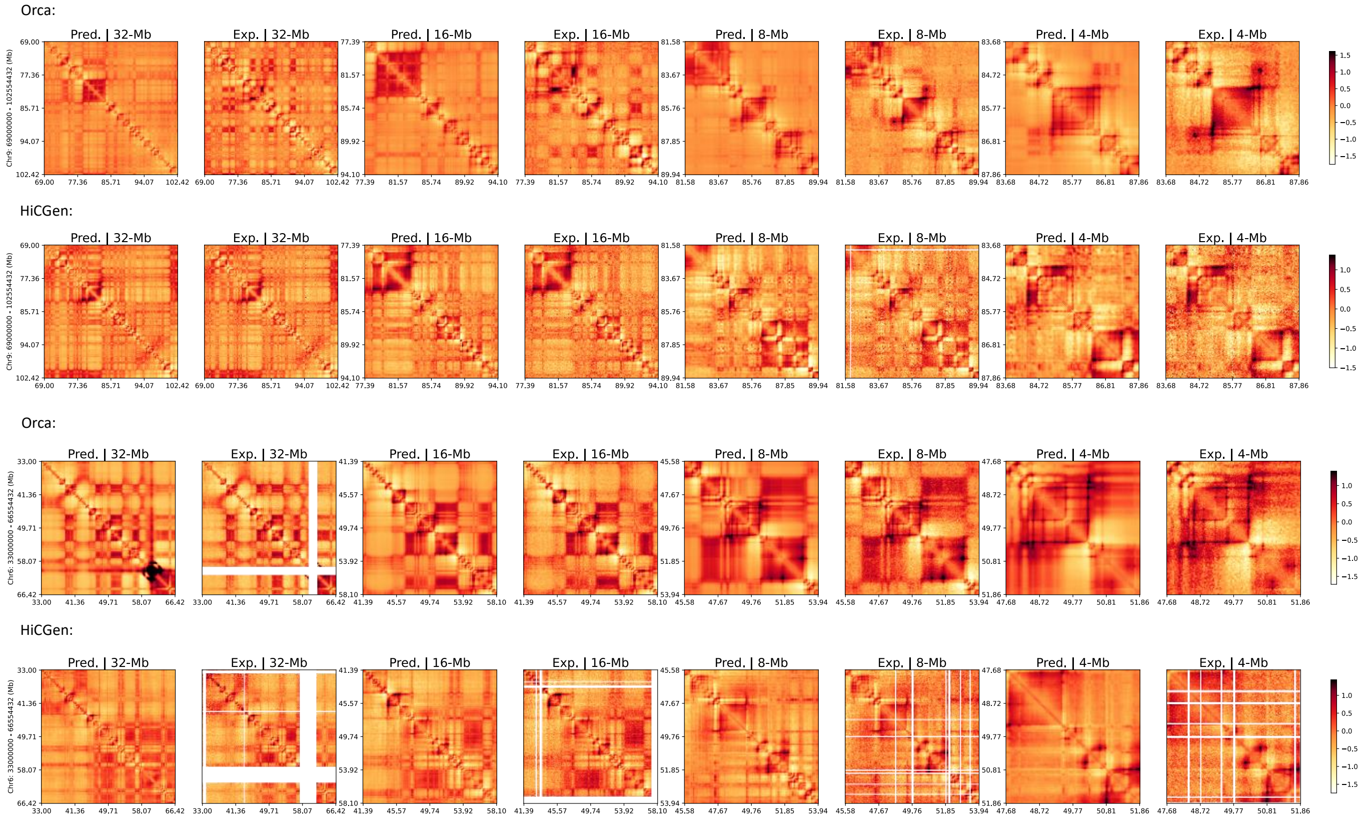

Supplementary Fig.3 | Example outputs of cell-type-specific predictions from Orca, C.Origami and HiCGen. a, Random selected de novo predictions of HCT116 contact matrices from C.Origami and HiCGen. The C.Origami model was trained with IMR90, while the HiCGen model was trained with GM12878. **b,** Comparisons of randomly selected predictions of Orca trained with HFF cell line and HiCGen trained with GM12878 at 128-kb, 64-kb, 32-kb and 16-kb resolutions.

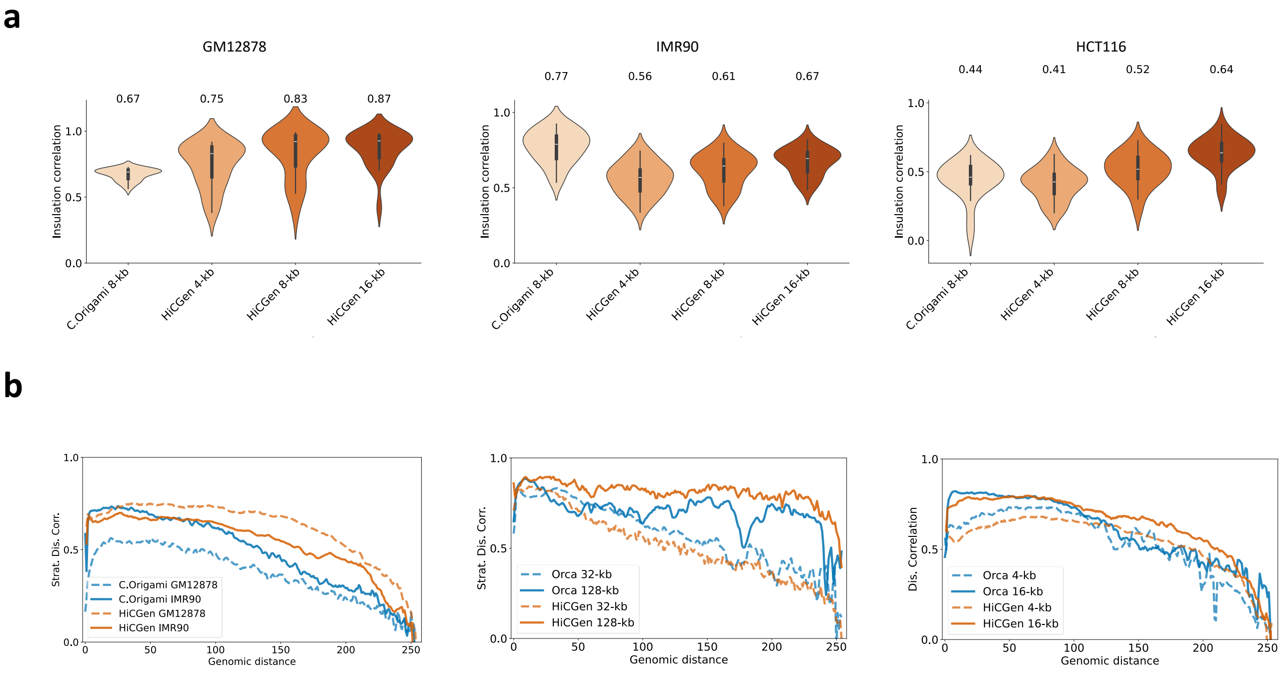

Supplementary Fig.4 | Genome-wide comparison of model performance on correlation metrics. a, correlations in insulation scores at TAD boundaries for predictions from each model output (C.Origami and HiCGen at 4-kb; HiCGen at 8-kb and HiCGen at 16-kb). Texts indicate the mean correlation coefficients for each cell lines. **b**, distance-stratified correlations of test set chromosome from each model output (HiCGen and Orca at 4-kb; C.Origami and HiCGen at 8-kb; HiCGen and Orca at 16-kb; HiCGen and Orca at 32-kb; HiCGen and Orca at 128-kb). The C.Origami model used for prediction was trained with IMR90 cell line. The HiCGen model used for prediction was trained with GM12878 cell line. The Oca model used for prediction was trained with HFF cell line.

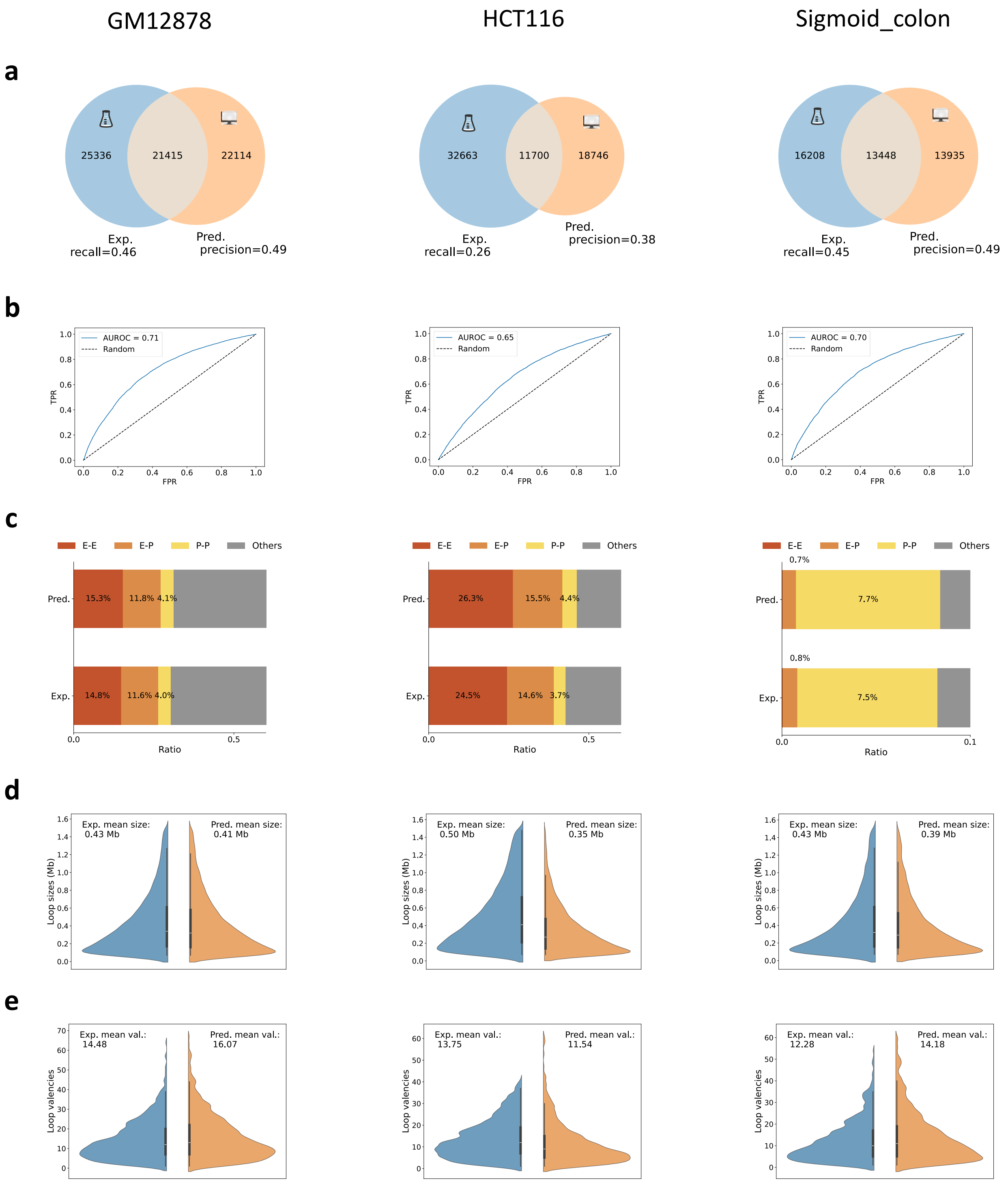

Supplementary Fig.5 | Genome-wide comparison of model performance on loop detection in GM12878, HCT116 and sigmoid colon cells. a, Venn diagrams of all loops called in experiments and predictions. The recall and precision of the predictions are also presented. **b,** ROC curves and the corresponding AUROC of the detected loops. **c,** Ratios of loops in three different categories, namely enhancer-enhancer (E-E), enhancer-promoter (E-P) and promoter-promoter (P-P) loops. **d,e,** Distributions of loop sizes (**d**) and loop valencies (**e**) for loops less than 1.5-Mb detected in Hi-C experiments and HiCGen predictions. Loop calling was conducted by Peakachu, with normalized contact map scaled to 10-kb-resolution.

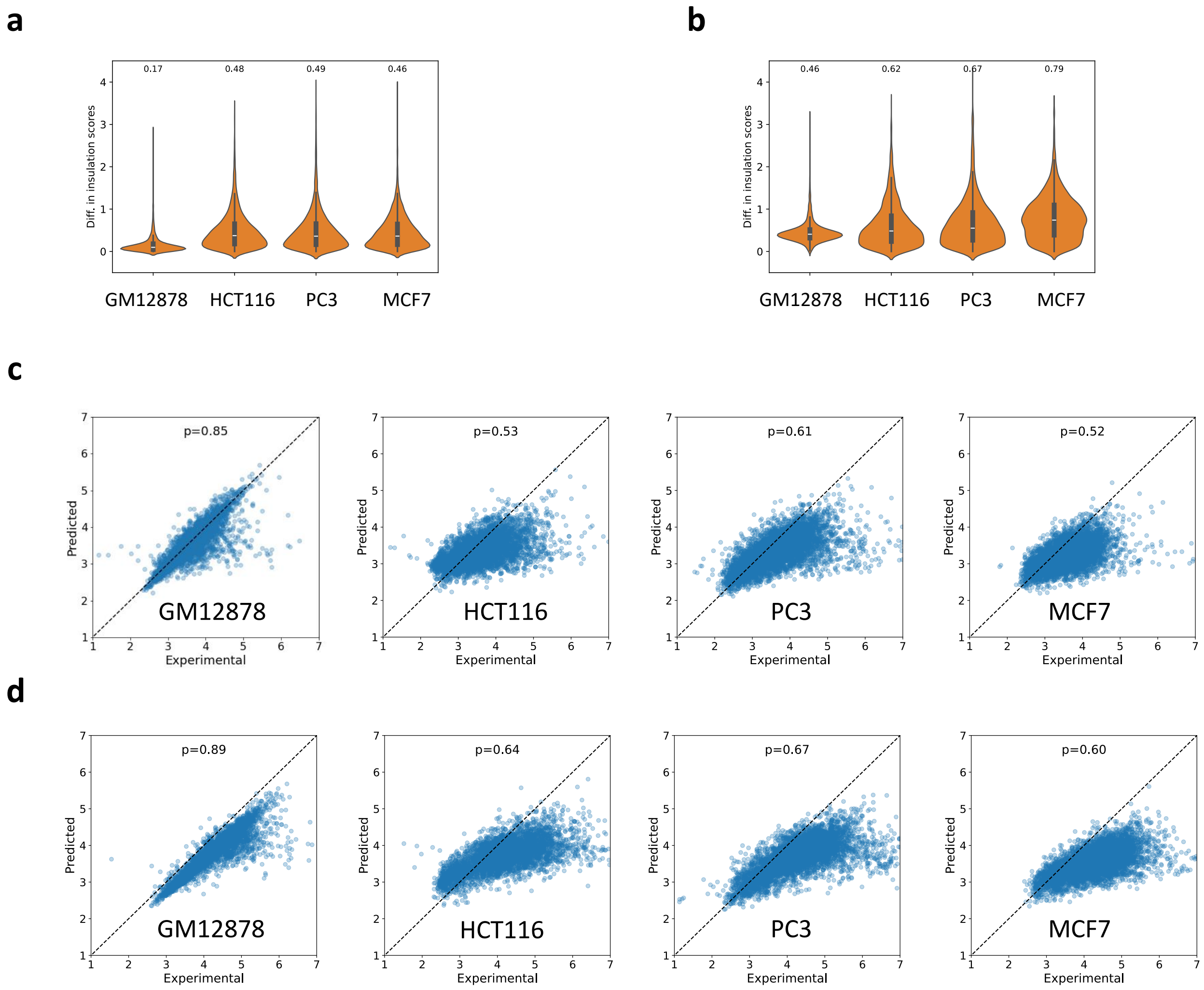

Supplementary Fig.6 | Genome-wide comparison of model performance on recapitulating insulation boundaries in GM12878, HCT116, PC3 and MCF7 cell lines. a,b, Difference in insulation scores at TAD boundaries determined from Hi-C matrices and from HiCGen perditions at 8-kb (**a**) and 16-kb (**b**) resolutions. Texts on top indicate the mean differences for each cell lines. **c,d,** 2D comparisons of insulation scores at TAD boundaries determined from Hi-C matrices and from HiCGen perditions at 8-kb (**c**) and 16-kb (**d**) resolutions. Texts on top indicate the Pearson correlation coefficients between experimental and predicted data for each cell lines. TAD boundaries for the normalized Hi-C contact map were identified by FAN-C. The 4-Mb model used for prediction was trained with GM12878 cell line.

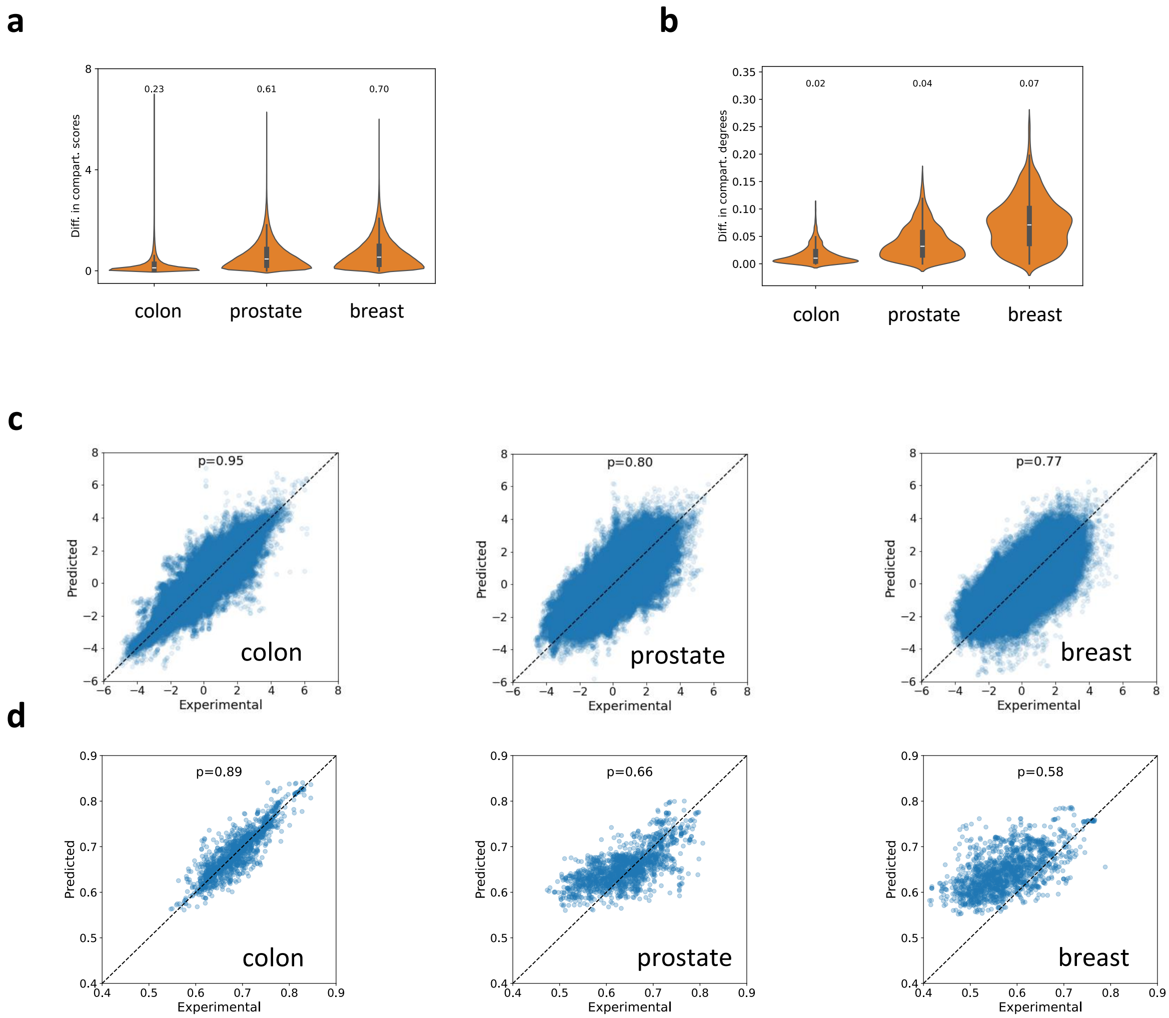

Supplementary Fig.7 | Genome-wide comparison of model performance on recapitulating compartments and compartmentalization in sigmoid colon, prostate gland and breast epithelium. a,b, Difference in compartment scores (**a**) and compartmentalization degrees (**b**) determined from Hi-C matrices and from HiCGen perditions at 128-kb-resolution. Texts on top indicate the mean differences for each cell types. **c,d,** 2D comparisons of compartment scores (**c**) and compartmentalization degrees (**d**) determined from Hi-C matrices and from HiCGen perditions at 128-kb-resolution. Texts on top indicate the Pearson correlation coefficients between experimental and predicted data for each cell types. The 32-Mb model used for prediction was trained with sigmoid colon.

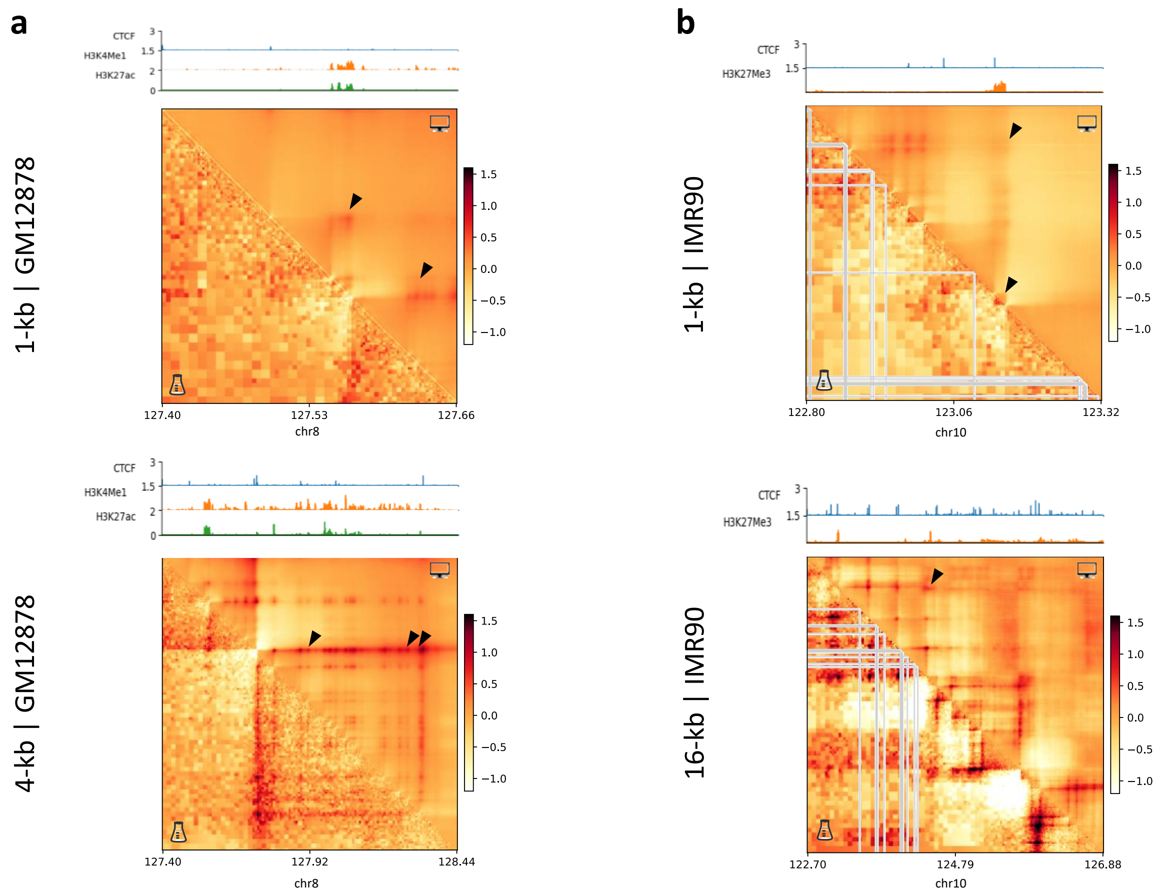

Supplementary Fig.8 | Prediction of protein-mediated and promoter-enhancer loops. a, Exemplary predictions of promoter-enhancer interactions and enhancer-enhancer interactions for selected genomic regions of GM12878 at 1-kb and 4-kb resolutions. **b,** Exemplary predictions of Polycomb-mediated interactions for selected genomic regions of IMR90 at 4-kb and 16-kb resolutions. Promoter-enhancer and enhancer-enhancer interactions are positioned by H3K4me1 and H3K27ac peaks, while Polycomb-mediated interactions are determined by H3K27me3 peaks. The predicted interactions that are faint or absent in experimental data are marked by black triangles.

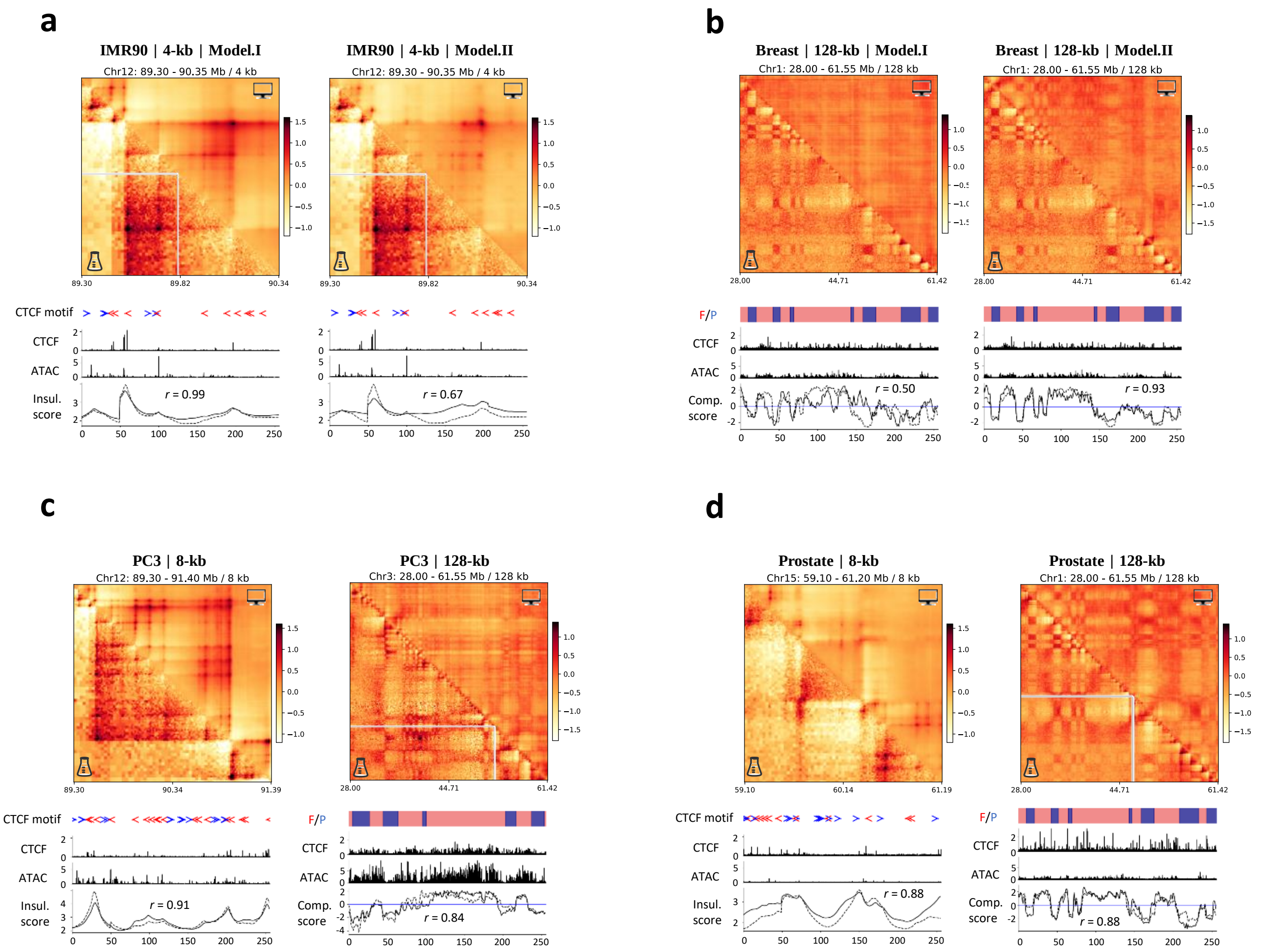

Supplementary Fig.9 | Extended predictions of the hierarchical contacts for various cell types. a, Comparison of predicted (upper triangular matrices) and experimental (lower triangular matrices) contact maps for a selected genome region of IMR90 at 4-kb resolution, utilizing Model I (trained on GM12878 data) and Model II (trained on sigmoid colon data). The contact matrices generated by Model I exhibit significantly higher accuracy compared to those generated by Model II, with respective Pearson correlation coefficients of the experimental (dashed lines) and predicted (solid lines) insulation scores 0.99 and 0.67 for Model I and II. **b,** Comparison of predictions for a selected genome region of breast epithelium at 128-kb resolution based on Model I and Model II. In this case, Model II gives a higher Pearson correlation coefficient of the compartment score of (0.93) compared to that for Model I (0.50). **c,d,** Exemplary predictions of contact maps, along with corresponding epigenetic profiles, insulation scores and compartment scores, for PC3 (**c**) and prostate gland (**d**) at 8-kb and 128-kb resolutions.

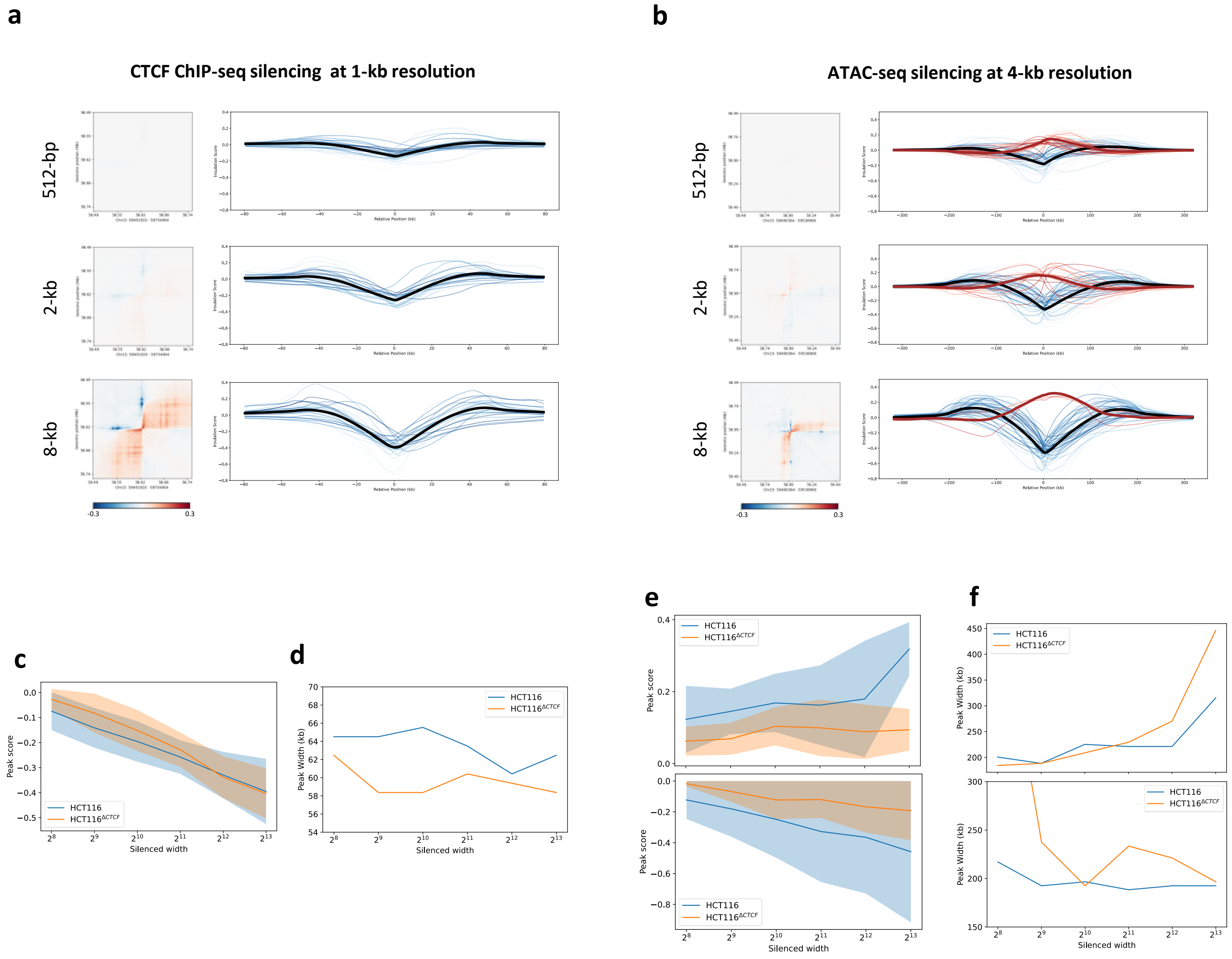

Supplementary Fig.10 | Range effect of genome perturbation at loop/TAD scale via editing CTCF ChIP-seq and ATAC-seq inputs. a, Difference in contact matrices and impact scores at 1-kb-resolution by silencing 512-bp, 2-kb or 8-kb CTCF ChIP-seq signals for HCT116. **b,** Difference in contact matrices and impact scores at 4-kb-resolution by silencing 512-bp, 2-kb or 8-kb ATAC-seq signals for HCT116. The top 30 highest impact scores (30-HIS) curves are respectively presented in (**a**) for CTCF-dominated and in (**b**) for ATAC-dominated fragments. Bold lines denote the average of 30-HIS curves with either enhanced (red) or attenuated (black) insulation scores. **c,d,** the dependence of the average peak value of the difference in insulation scores (**c**) and peak width (**d**) of 30-HIS curves at 1-kb-resolution on the silenced width of the CTCF ChIP-seq signals within CTCF-dominated regions. While the peak value increase with the silenced width, the peak width fluctuates around 62 kb. Silencing CTCF ChIP-seq signals has a slightly wider range impact (~4 kb) on HCT116 genome than that on HCT116$^{\Delta CTCF}$. **e,f,** the dependence of average peak value (**e**) and peak width (**f**) of 30-HIS at 4-kb-resolution on the silenced width of the ATAC-seq signals within ATAC-dominated regions. A larger peak value is obtained for HCT116 than that for HCT116$^{\Delta CTCF}$. The peak width increases with the width of the silenced signals for positive peaks (top), while it fluctuates around 200 kb for negative peaks (bottom). Notably, the impact width of silencing either CTCF or ATAC signals on insulation-reduced fragments remains consistent regardless of the length of the silenced region, whereas silencing ATAC-seq signals results in broader changes to insulation-enhanced fragments as the silenced fragment length increases.

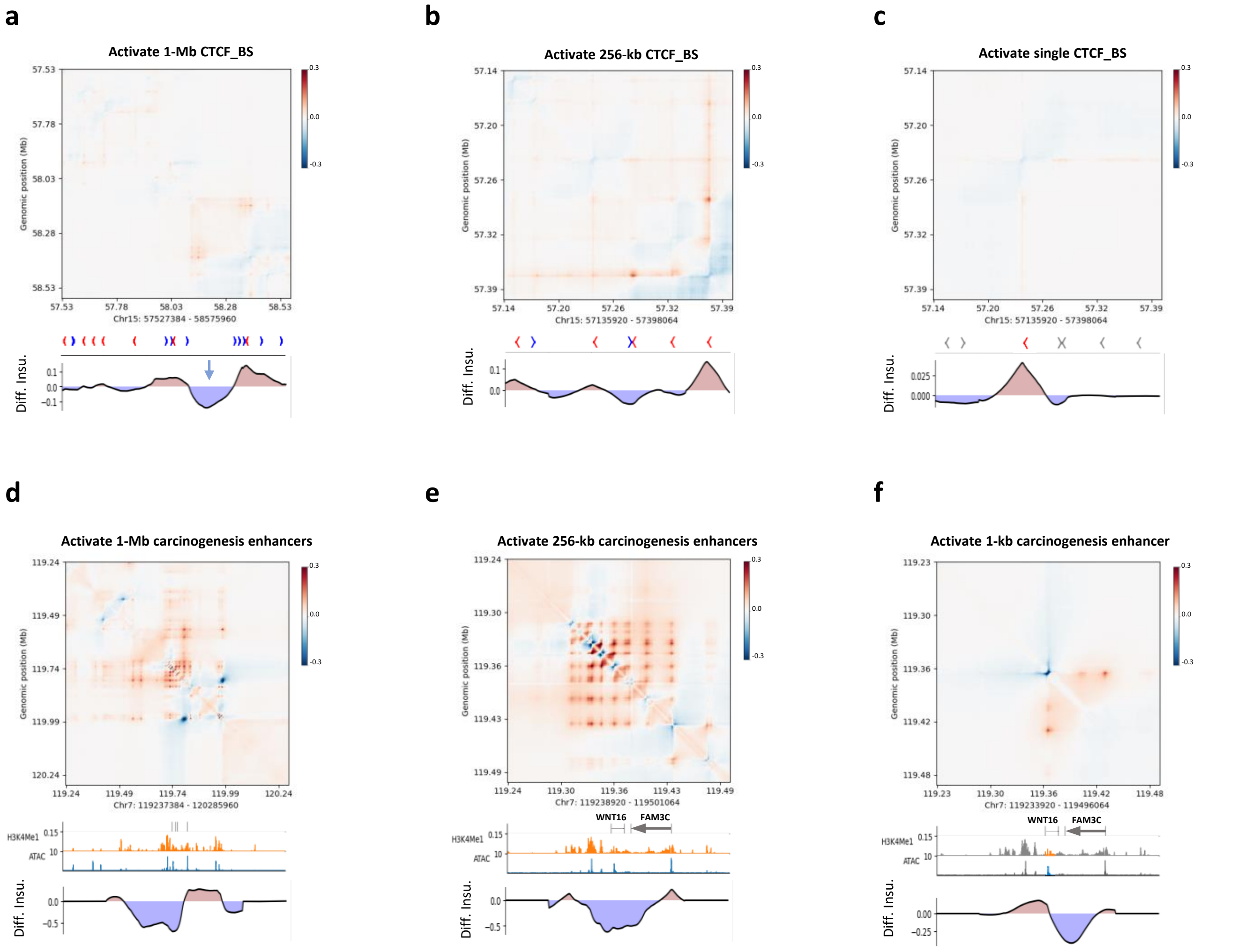

Supplementary Fig.11 | In silico perturbation of the genome at loop/TAD scale through activating CTCF ChIP-seq and ATAC-seq signals. a-c, Difference in contact matrices and insulation scores at 4-kb (**a**) and 1-kb (**b**,**c**) resolutions by activating multiple CTCF binding sites (CTCF-BS) within 1-Mb (**a**) or 256-kb (**b**) genome, or single CTCF-BS (**c**) for GM12878. Variations of the insulation scores are dependent on the orientation of CTCF motifs. For instance, the intra-contacts for genome containing a set of inward-oriented CTCF-BS are enhanced (blue arrow in **a**). **d-f,** Difference in contact matrices and insulation scores at 4-kb (**d**) and 1-kb (**e**,**f**) resolutions by activating ATAC-seq signals within H3K4me1 peak regions of HCT116 for 1-Mb (**d**), 256-kb (**e**) or 1-kb (**f**) genome of sigmoid colon. Two markedly upregulated genes, WNT16 and FAM3C, are situated within genomic regions exhibiting elevated intra-chromosomal contacts. As illustrated in (**f**), the activation of a single nearby enhancer adjacent to WNT16 is sufficient to enhance its interaction with the promoter region of FAM3C.

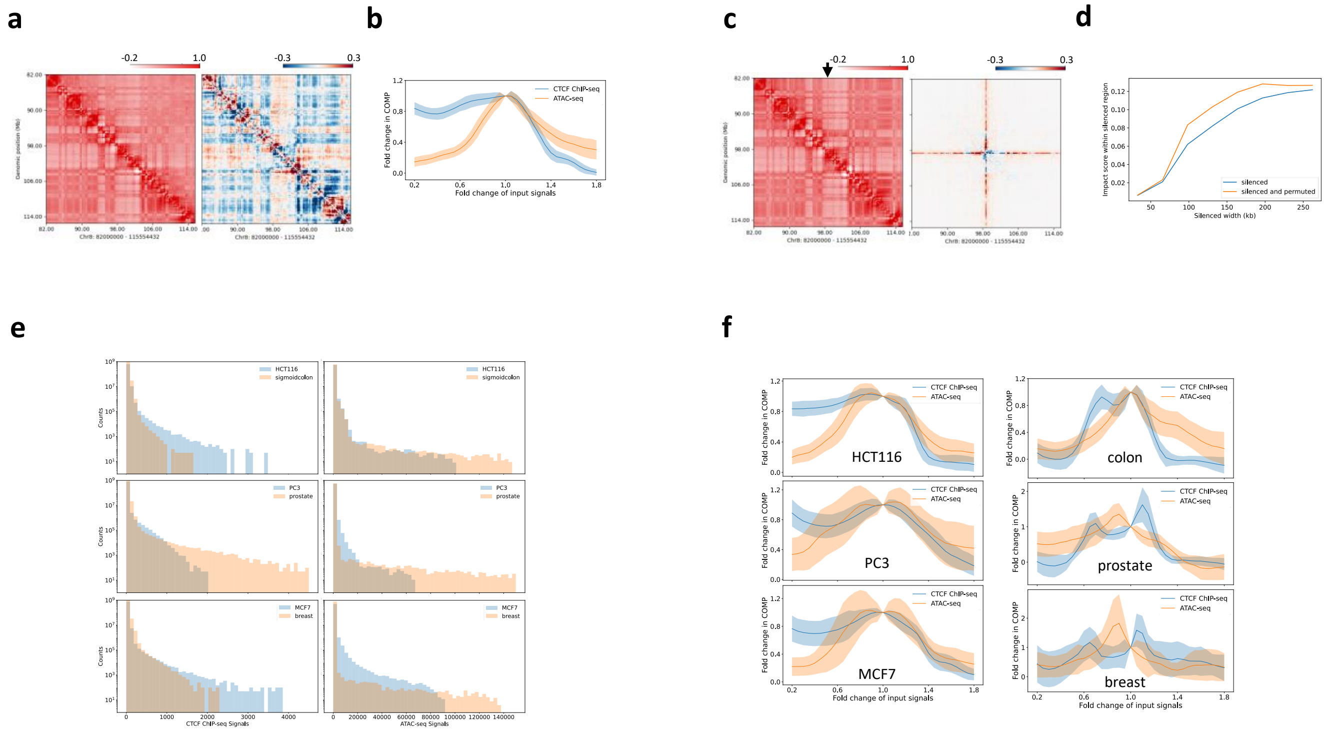

Supplementary Fig.12 | In silico perturbation of the genome at compartment scale for various cell types. a, Comparison of the contact maps prior to (lower left) and subsequent to (upper right) 1.2-fold increase to the ATAC-seq signals for GM12878, along with the difference map. **b,** The dependence of compartment scores on the fold-change factor of CTCF ChIP-seq and ATAC-seq signals for GM12878. **c,** Comparison of the contact maps prior to (lower left) and subsequent to (upper right) the silencing of 128-kb ATAC-seq signals within sub-compartment A2 of GM12878, along with the difference map. **d,** Comparison of impact scores with or without permuting the silenced sequence based on 16-bp segments, at various widths of the silencing region of the ATAC-seq signals. **e,** Comparison of the distributions of CTCF ChIP-seq and ATAC-seq signals for various normal and cancerous cell lines. **f,** The fold-change of compartment scores as a function of the fold-change factor of CTCF ChIP-seq or ATAC-seq signals for various normal and cancerous cell lines.

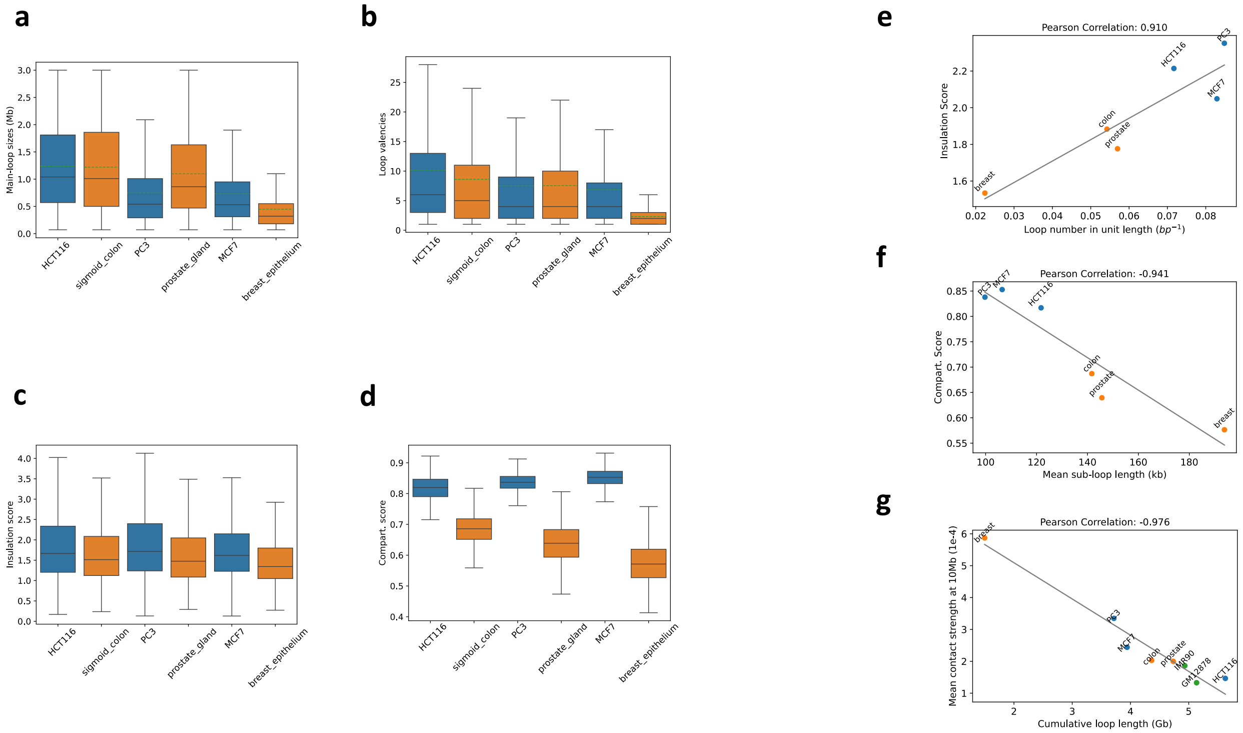

Supplementary Fig.13 | Correlation of the genome organizations at different scales for various cell types. a,b, Box plots of the distribution of root-loop sizes (**a**) and loop valencies (**b**) for cancerous (blue) and normal (orange) cells. The mean values are denoted by green dashed lines. **c,d,** Box plot of the distribution of genome-wide insulation scores at TAD boundaries (**c**) and compartmentalization scores (**d**) for cancerous and normal cells. **e-g,** Correlations between insulation scores and the number of loops in unit length (**e**), correlations between compartmentalization degree and mean sub-loop length (**f**), and correlations between contact strength at 10 Mb and cumulative loop length (**g**). Large Pearson coefficients (0.91, -0.94, and -0.98) are obtained for all these correlations.

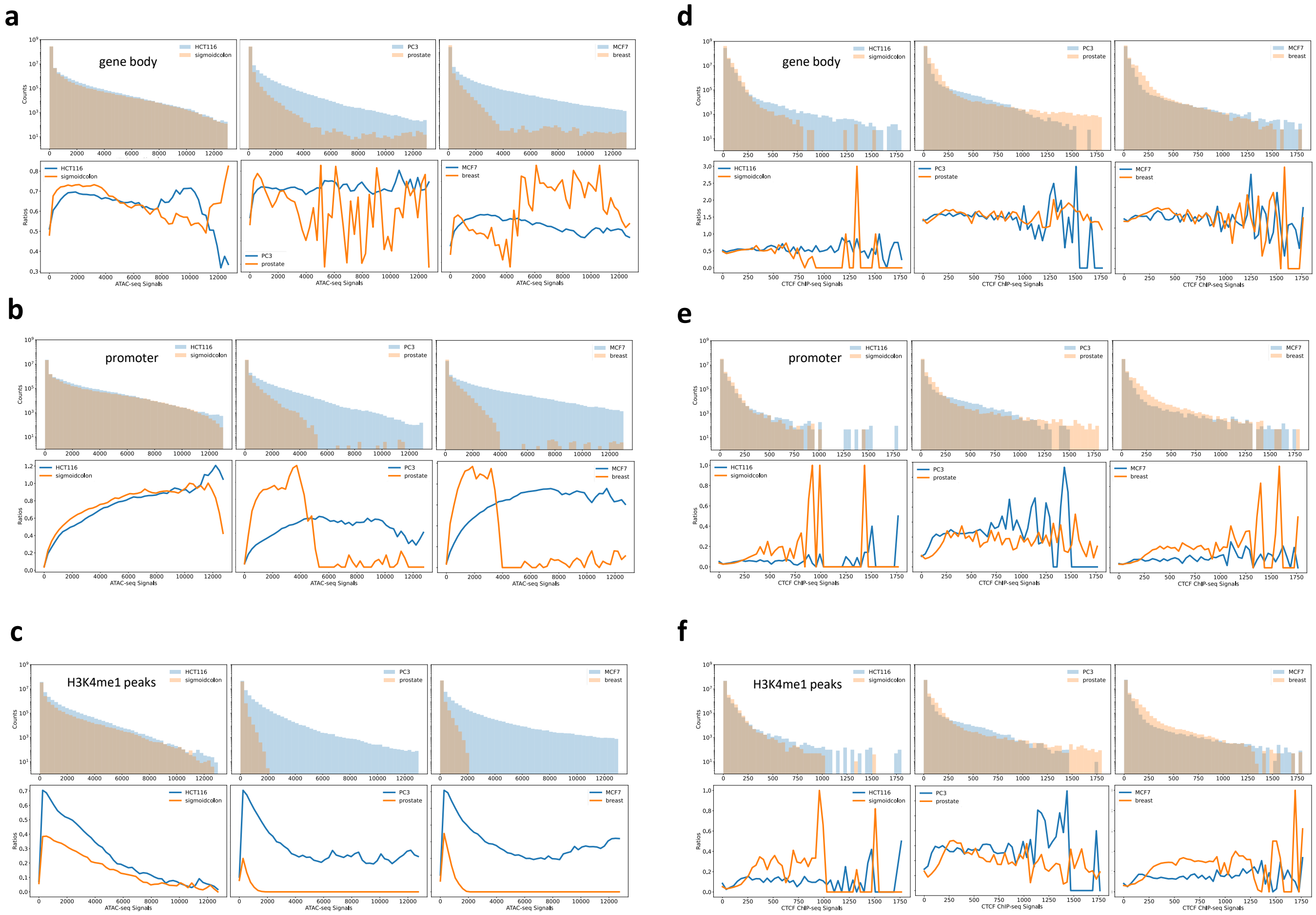

Supplementary Fig.14 | Distributions of ATAC-seq and CTCF ChIP-seq signals at functional regions in cancerous and normal cells. a-c, Distributions of ATAC-seq signals (top) and the their ratio to total ATAC-seq signals (bottom) at gene bodies (**a**), promoters (**b**) and enhancers (**c**) for various cancerous and normal cells. **d-f,** Distributions of CTCF ChIP-seq signals and their ratio to total CTCF ChIP-seq signals at gene bodies (**d**), promoters (**e**) and H3K4me1 peaks (**f**) for various cancerous and normal cells. Notably, the primary differences between cancerous and normal cells are observed in ATAC-seq signals within regions of H3K4me1 peaks: cancerous cell lines exhibit significantly higher ratios to total ATAC-seq signals compared to normal cells.

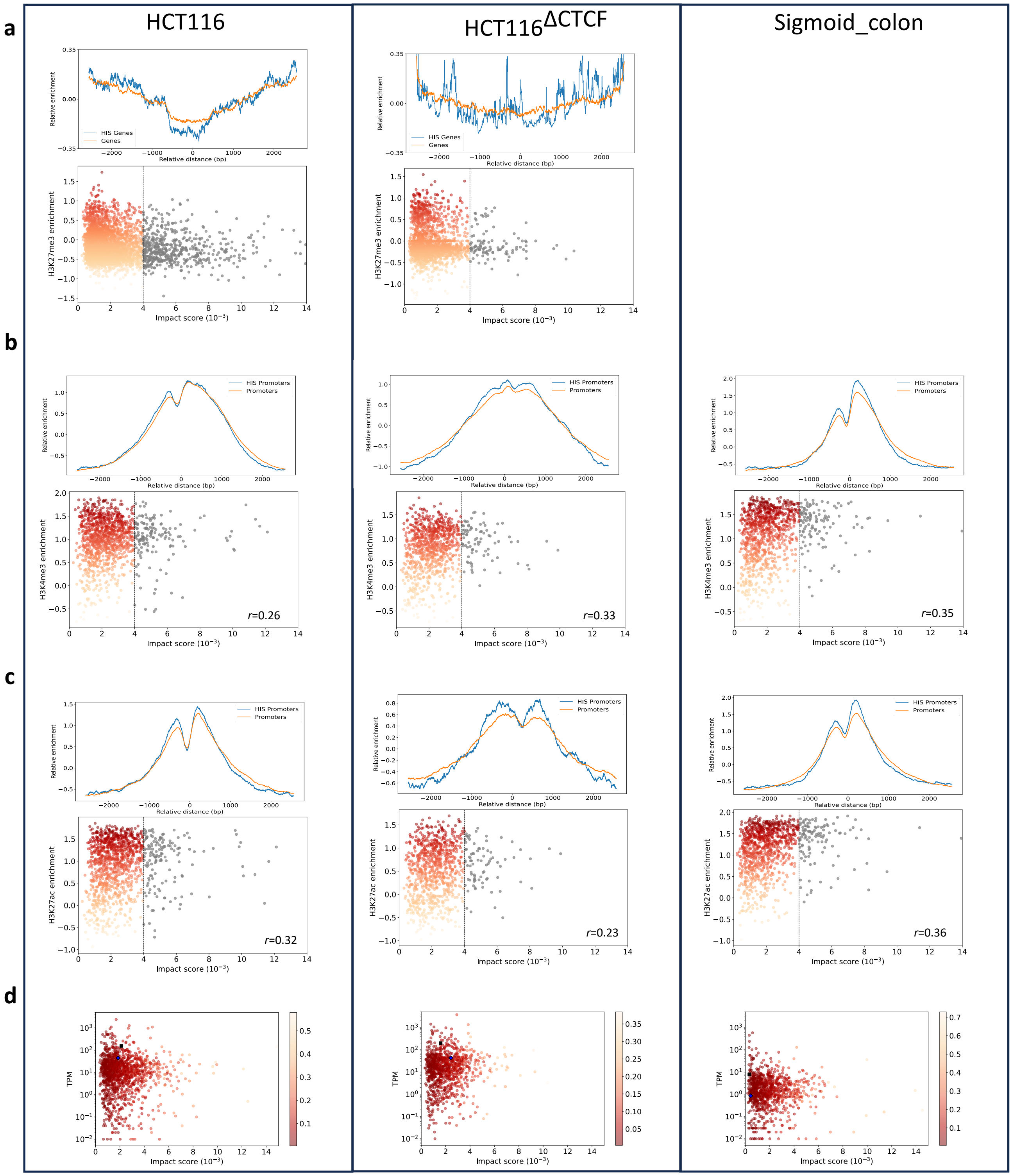

Supplementary Fig.15 | Relative enrichment of epigenetic signals and corresponding gene expression for promoters in ATAC-dominated group. a, Z-score normalized relative enrichment (upper panel) of H3K27me3 signals, and their correlation with impact scores (lower panel) by silencing 1-kb ATAC-seq signals in ATAC-dominated gene bodies. Orange and blue lines respectively denote enrichment curves for all gene bodies and gene bodies with impact score larger than 0.004. **b,c,** Relative enrichment of H3K4me3 (**b**) and H3K27ac (**c**) signals in proximity to transcription start site (TSS), and their correlation with impact scores by silencing 1-kb ATAC-seq signals in ATAC-dominated promoters. **d,** Transcripts per million (TPM) as a function of impact score by silencing 1-kb ATAC-seq signals in ATAC-dominated promoters. The color-bar indicates the peak value of the insulation peak induced by silencing the promoter. Blue and black squares respectively indicate TSS of TP53 and MYC.

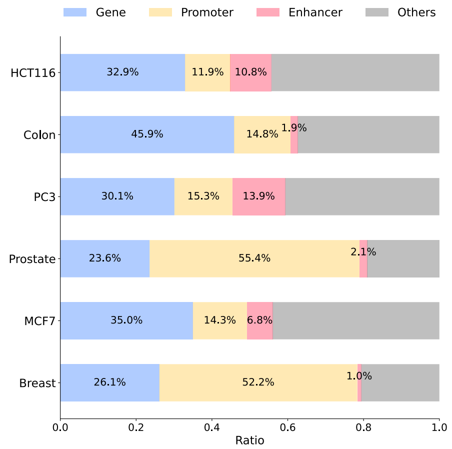

Supplementary Fig.16 | Distribution of segment categories within HIS-subset in cancer and normal cells. Distributions of segment categories for all fragments with high impact scores (HIS-subset) upon silencing of 1-kb ATAC-seq signals.

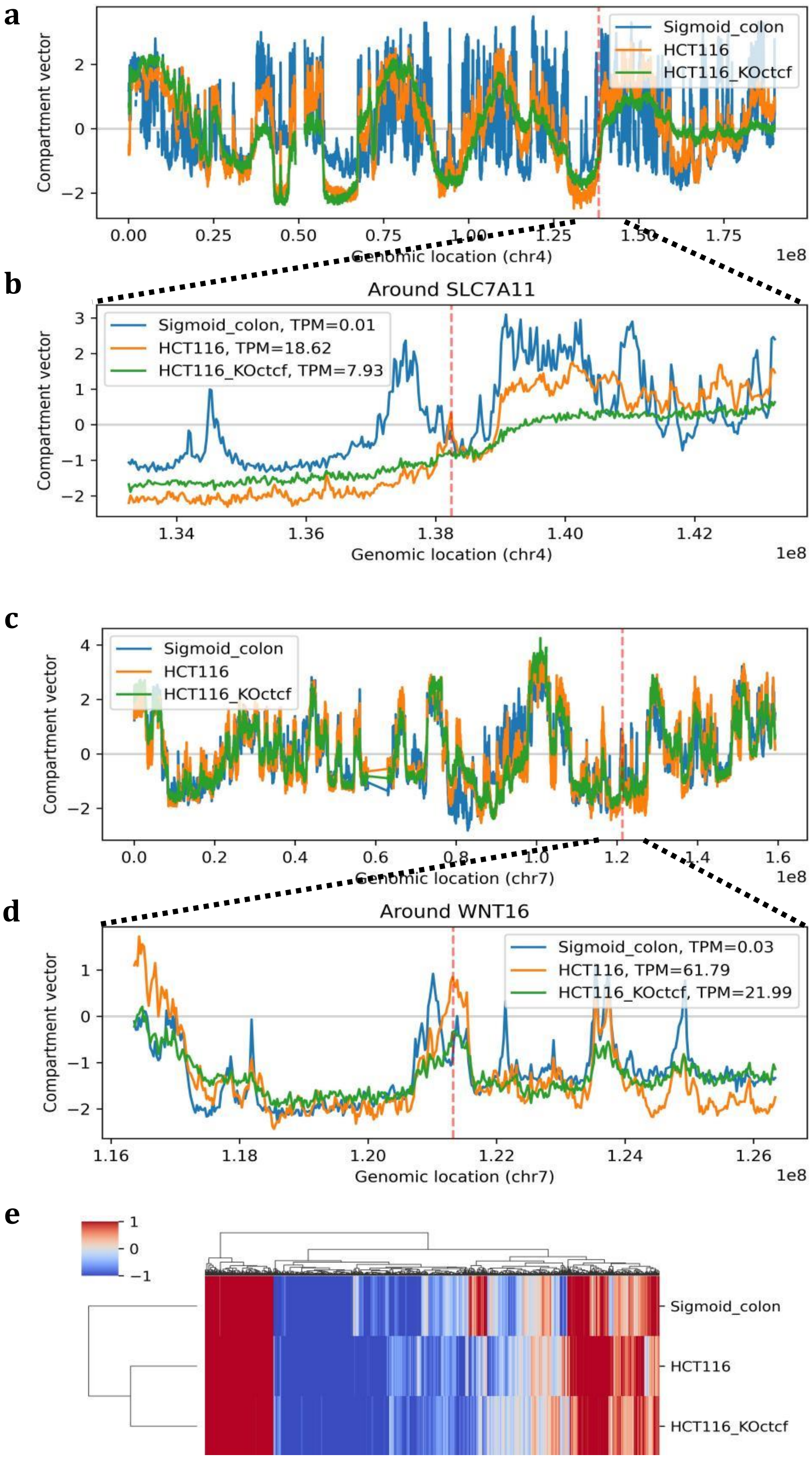

Supplementary Fig.17 | Compartment scores and hierarchical clustering for compartment vectors. a-d, Compartment scores for chr4 (**a**), selected fragments around chr4: SLC7A11 (**b**), chr7 (**c**) and selected fragments around chr7: WNT16 (**d**). Compartments flip from B to A in proximity to SLC7A11 and WNT16 for HCT116, compared to those for sigmoid colon. **e,** Hierarchical clustering for compartment scores of all bins of chr4 and chr7. Each column represents a 25-kb bin and each row represent a cell sample. Pixel colors indicate score values.

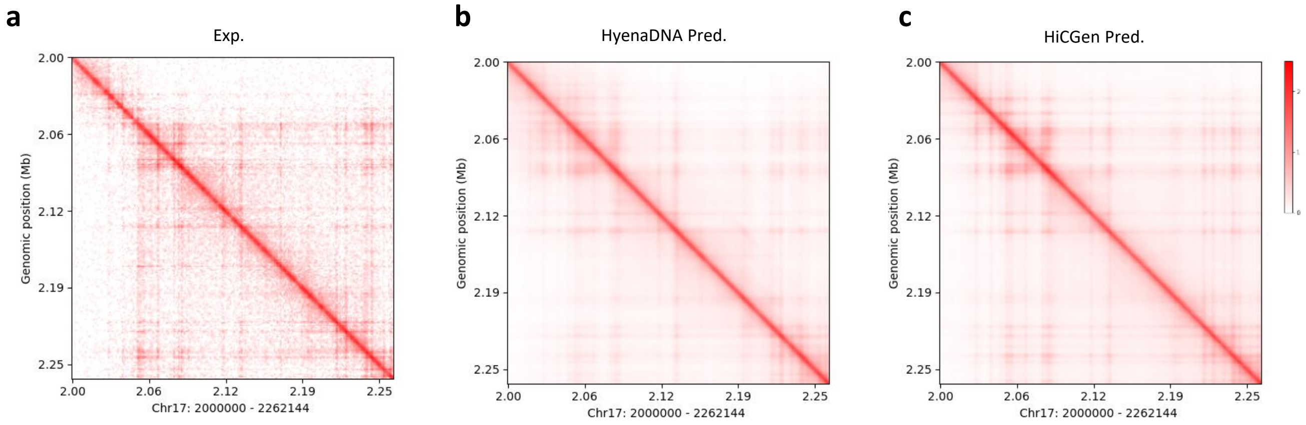

Supplementary Fig.18 | Comparison of predictions of models with different architectures at 1-kb-resolution. Predictions of model based on HyenaDNA (b) and HiCGen (c) are compared with Hi-C contact matrices (a) of randomly selected 256-kb genome of GM12878.

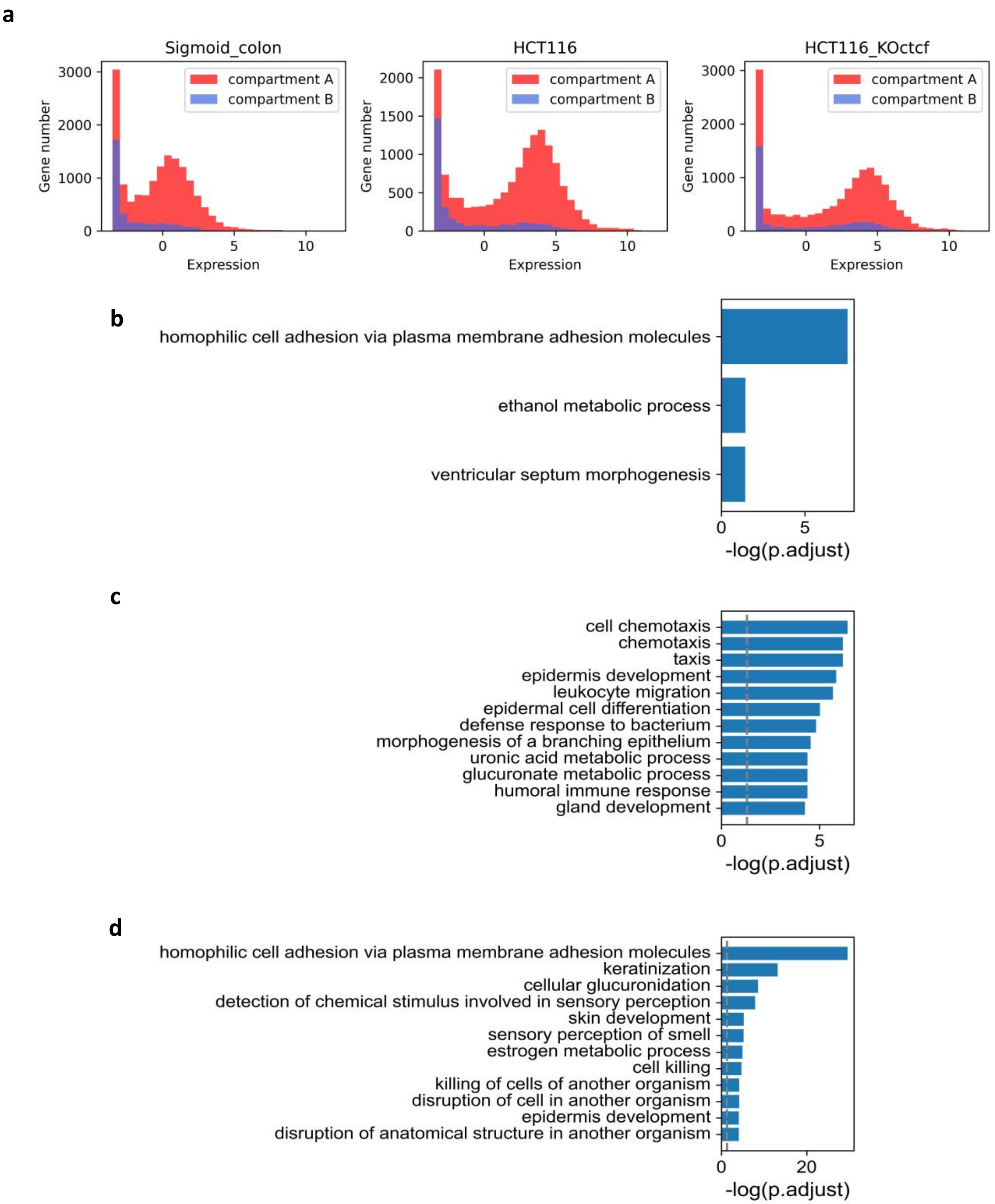

Supplementary Fig.19 | Distributions of expression levels and GO functional enrichment for genes that experience compartment flipping in sigmoid colon, HCT116 and HCT116$^{\Delta CTCF}$ a, Distributions of expression level (log2(TPM+0.1)) for genes in compartments A and B. **b,c,** From sigmoid colon to HCT116, GO functional enrichment for genes that switch from compartment A to B (**b**) and genes switch from compartment from B to A (**c**). **d,** From HCT116 to its CTCF knockout condition, GO functional enrichment for genes that switch from compartment B to A (no enrichment was obtained for genes that switch from compartment A to B).

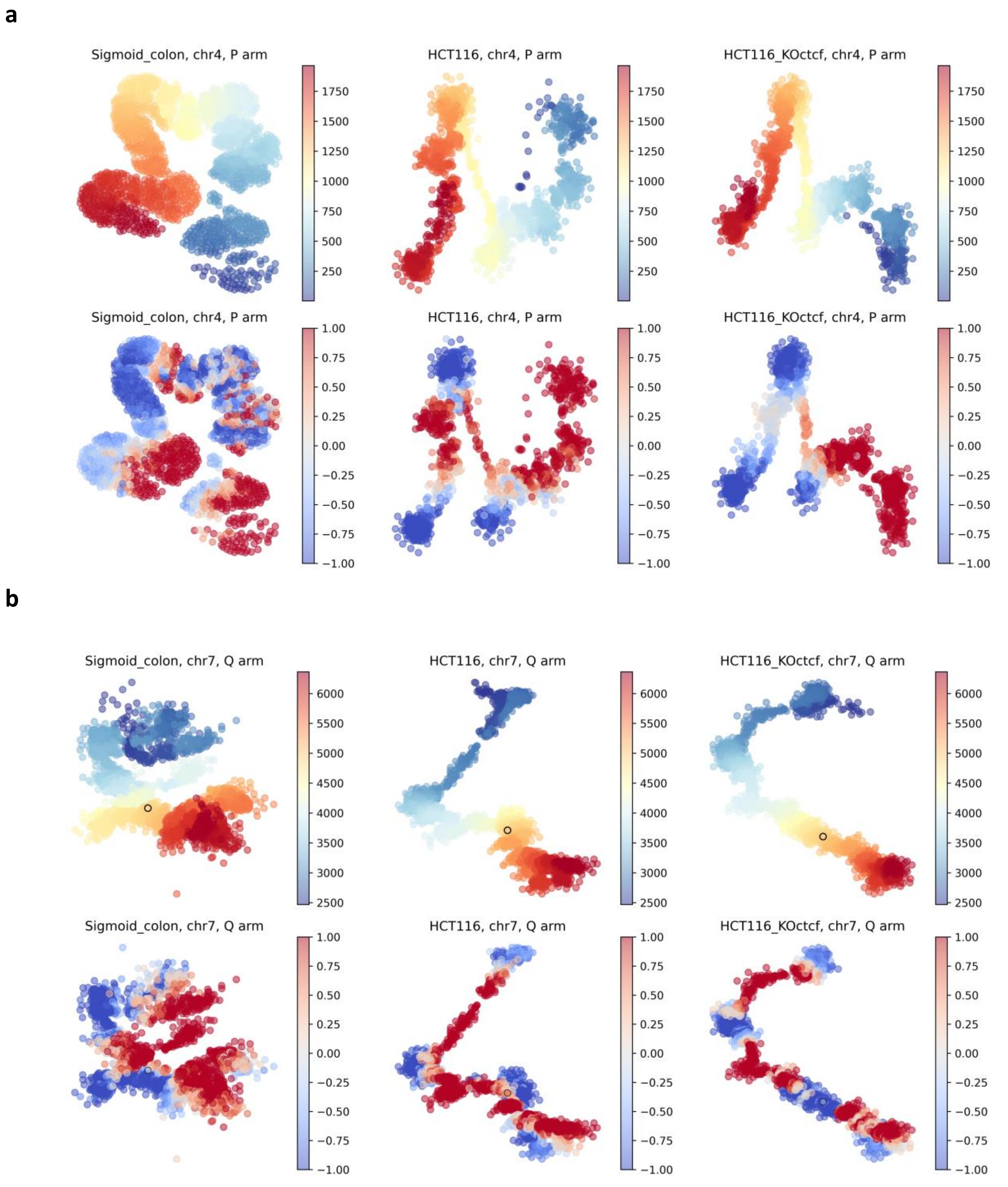

Supplementary Fig.20 | Two-dimensional layouts of average C_TG matrix. Force-directed layouts of C_TG distance matrix of P arm of chromosome 4 (**a**) and Q arm of chromosome 7 (**b**) in sigmoid colon, HCT116 and HCT116$^{\Delta CTCF}$. Each point represents a 25-kb bin. The upper panel is colored by sequence and the lower panel is colored by compartment score.

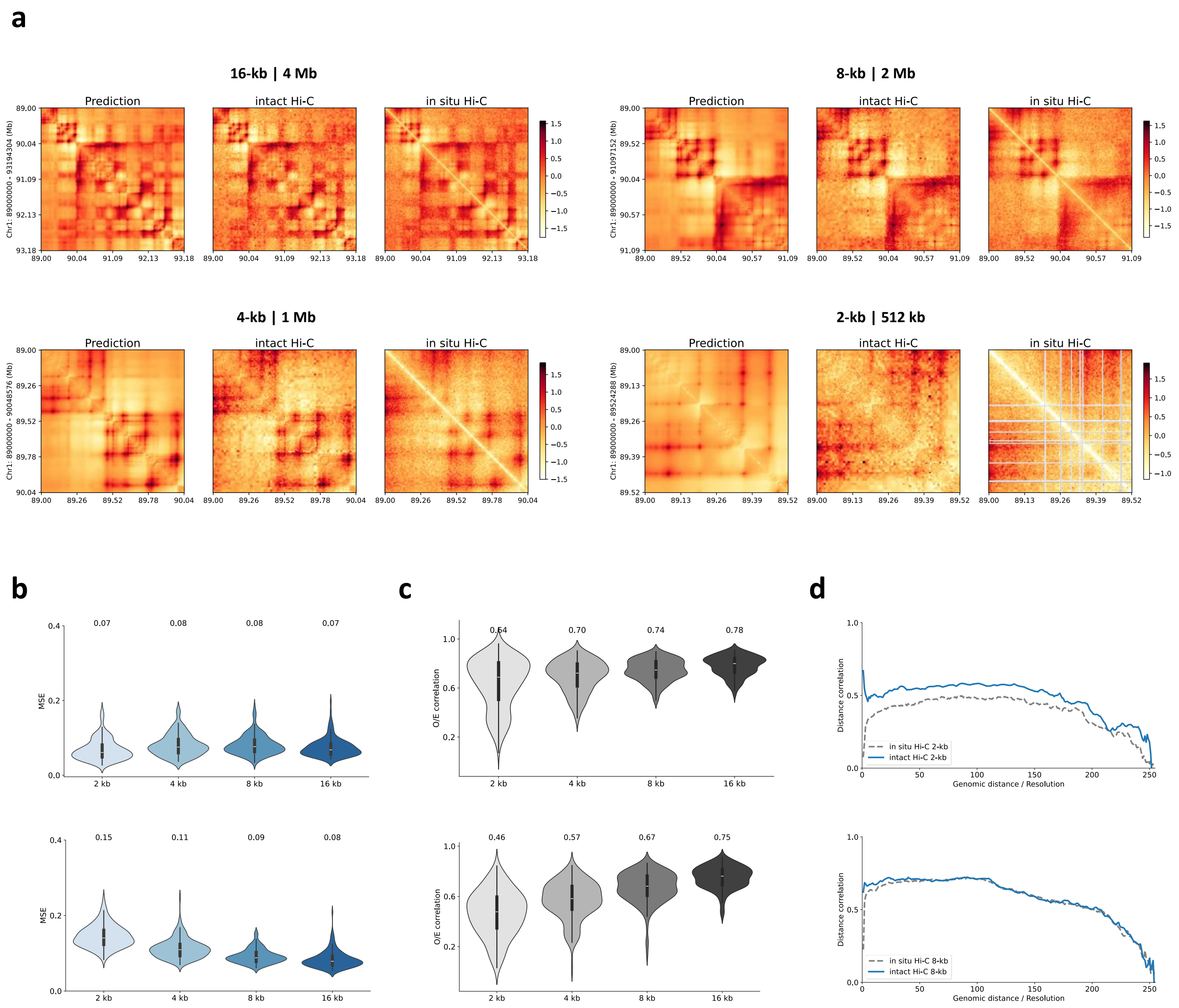

Supplementary Fig.21 | a, Comparisons between predicted matrices with intact Hi-C and in situ Hi-C results at 16-kb, 8-kb, 4-kb and 2-kb resolutions. **b,c,** Comparisons of mean squared error (b) and Pearson correlation coefficients (c) of predicted contacts for chr15 with respect to intact Hi-C (upper panel) and in situ Hi-C (lower panel) data. **d,** Distance stratified correlations between predicted and two sources of Hi-C data for test-set chromosome (chr15).
